# Effect of Magnetorheological Grease’s Viscosity to the Torque Performance in Magnetorheological Brake

**DOI:** 10.3390/ma15165717

**Published:** 2022-08-19

**Authors:** Khairul Anwar Abdul Kadir, Nurhazimah Nazmi, Norzilawati Mohamad, Muhammad Kashfi Shabdin, Dimas Adiputra, Saiful Amri Mazlan, Nur Azmah Nordin, Shahir Mohd Yusuf, Ubaidillah

**Affiliations:** 1 Engineering Materials and Structures (eMast) iKohza, Malaysia-Japan International Institute of Technology (MJIIT), Universiti Teknologi Malaysia, Jalan Sultan Yahya Petra, Kuala Lumpur 54100, Malaysia; 2 Faculty of Engineering, Universiti Malaysia Sabah, Jalan UMS, Kota Kinabalu 88400, Malaysia; 3 Department of Physics, Faculty of Science, Universiti Putra Malaysia, UPM Serdang, Selangor 43400, Malaysia; 4 Electrical Engineering Department, Institut Teknologi Telkom Surabaya, Surabaya 6023, Indonesia; 5 Mechanical Engineering Department, Universitas Sebelas Maret, J1. Ir. Sutami 36A, Kentingan, Surakarta 57126, Indonesia

**Keywords:** magnetorheological brake, braking torque, magnetorheological grease, additive, rheological properties

## Abstract

Recently, magnetorheological grease (MRG) has been utilized in magnetorheological (MR) brakes to generate a braking torque based on the current applied. However, the high initial viscosity of MRG has increased the off-state torque that led to the viscous drag of the brake. Therefore, in this study, the off-state viscosity of MRG can be reduced by the introduction of dilution oil as an additive. Three samples consist of pure MRG (MRG 1) and MRG with different types of dilution oil; hydraulic (MRG 2) and kerosene (MRG 3) were prepared by mixing grease and spherical carbonyl iron particles (CIP) using a mechanical stirrer. The rheological properties in the rotational mode were examined using a rheometer and the torque performances in MR brake were evaluated by changing the current of 0 A, 0.4 A, 0.8 A, and 1.2 A with fixed angular speed. The result shows that MRG 3 has the lowest viscosity which is almost 93% reduction while the viscosity of MRG 2 has lowered to 25%. However, the torque performances generated by MRG 3 were highest, 1.44 Nm, when 1.2 A of current was applied and followed by MRG 2 and MRG 1. This phenomenon indicated that the improvement of torque performances was dependent on the viscosity of MRG. By reducing the viscosity of MRG, the restriction on CIP to form chain formation has also decreased and strengthen the torque of MRG brake. Consequently, the utilization of dilution oil in MRG could be considered in MR brake in near future.

## 1. Introduction

Magnetorheological (MR) brakes have several potential applications in a variety of areas and machineries, including automotive, construction, fitness equipment, and computer numerical control machine tools. MR brakes have a simple design, a smooth and dependable functioning mechanism, a rapid and reversible reaction, and are easy to control in the presence of a magnetic field [1]. Carlson et al. [2] presented the first MR brake actuator in 1998, using MR fluid (MRF) as a medium, and the highest produced torque was up to 4 Nm with an applied current of 1 A. This outcome was sufficient to be applied to low torque devices, such as ankle foot orthoses [3]. MRF has been utilized in dampers [4,5] and brakes [6] due to its unique ability to modify rheological properties by adjusting the strength of the magnetic field. As a result, the proposal of using MRF in car braking systems [7,8,9] has been presented, with a target torque of at least 500 Nm for the usage of 1000 kg automobile [10]. As a result, numerous ways to improving the torque performances of MR brakes for automotive applications have been adopted. Researchers proposed redesigning the disk-type MR brake [11,12], drum-type MR brake [7,8], and T-shape MR brake [13] by raising the external magnetic field strength [14] and enhancing the active fluid gap in the MR brake [7,15]. Furthermore, previous research has shown that the rheological properties of carrier fluid, particularly MRF, have an effect on the torque performances of the MR brake. It has been proven that increasing the weight percentages (wt%) of magnetic particles [16] and the addition of additives [17] could enhance the rheological properties of MRF subsequently increased the torque performance in MR brake. Aside from that, commercial MRF has a high viscosity, resulting in high off-state (no magnetic field) torque and low power consumption [18]. Nonetheless, using MRF has a few drawbacks, such as leakage and sedimentation issues, which degraded their performance for long-term operations [19]. Therefore, MR grease (MRG) has been introduced and suggested to overcome the problem by using highly viscous grease as a carrier fluid [20,21]. The operating temperature, on the other hand, is one of the elements influencing MRG performance. Mohamad et al. [22] investigated this event in their thermal investigation in terms of dropping point. According to their research, the true limit of the temperature grease to be used as a lubricant in the semi-solid state to preserve its structure before losing consistency of plasticity was 199 °C. Another study performed by Wang et al. [23] found that the MR effect or the change in mechanical properties of MRG hit 90,328% at 70 °C in the response to the magnetic field. The finding has demonstrated that increasing the temperature has affected the modulus of the MRG effectively, which is crucial for the practical application of MRG

There have been a few studies on the torque transfer application of MRG, particularly in MR clutches and MR brakes. Gordaninejad et al. [24] compared the braking torque performance of MRG with a different wt% of carbonyl iron particle (CIP) with MRF under various operating speeds from 300 to 1200 rpm in MR clutch. They discovered that when the wt% of CIP increased, so did the torque create by MRG. The initial torque of MR clutch using MRG with 99 wt% of CIP was higher by 200% compared to the same clutch using commercial MRF at off-state condition. Furthermore, in the on-state situation (the existence of a magnetic field), the torque generated by MRG was 75% greater than MRF. However, the obtained findings were incomparable because the wt% of CIP in MRF was lower than in MRG. Then, Sukhwani et al. [25] extended the research in MR brake by using an equal wt% of CIP, 90% in MRG and MRF The discovery indicated that MRG’s first braking power output was two times greater than MRF’s under off-state conditions. However, at on-state condition, the brake power output of MRG was two times lower than MRF. Thus, to enhance the torque performance of MRG, Singh et al. [26] have designed a new wedge-shaped drum MR clutch by utilizing 50 and 75 wt% of CIP in MRG. The obtained torque has increased by 150% higher than the traditional MR clutch. However, the off-state torque of MRG has increased nearly 8% as the CIP content increased from 50 to 75 wt%. This phenomenon was presumably caused by the impacts of MRG rheological properties on torque performance in MR brake. Thus, Dai et al. [19] explored the effect of wt% on the rheological properties and their performance in speed reduction of MR brake. The viscosity, shear stress, and yield stress of MRG have reduced with increasing the wt% CIP at off-state condition. For the performance of MRG brake test, the initial rotational speed at off-state condition was fixed at 13,000 r/min. With the increased of the magnetic field, the rotational speed of MR brake was decreased for all samples. However, the rotational speed reduction produced by MRG with 50 wt% of CIP was higher than 30 and 10 wt% of CIP. Thus, the increment of wt% of CIP has enhanced the chain formation in MRG brake. However, the high initial viscosity of the carrier fluid was a disadvantage, and it should be significantly lowered to reduce the brake’s viscous drag in the off-state condition [27,28]. According to Nguyen and Choi [29] as the MR brake rotated, the high viscous drag created a large amount of heat energy due to the device’s zero-field friction. This problem became more severe when the MR brake was employed in device with high torque and power, which also increased the MR brake’s size. As a result, an effort has been taken in order to reduce the initial viscosity of MRG, such as by introducing the additives.

Previous study has shown that introducing dilution oil can reduce the initial viscosity of MRG. According to Kim et al. [30], the addition of 5 wt% kerosene oil reduced the initial viscosity of the MRG, indicating greater dispersion of magnetic particles in the grease medium. Then, Mohamad et al. [31] studied the compatibility of several oils (kerosene, hydraulic, and castor) toward MRG behavior. Interestingly, the result showed that the hydraulic oil was the most compatible with the grease medium compared to other types of dilution oil, and the initial apparent viscosity of the MRG decreased as increasing the amount of dilution oil. This event happened owing to the presence of oils, which reacted as a lubricant in the MRG and increased the dispersion of CIP in the grease medium. In another study, the influence of oil percentages, notably silicone oil and castor oil as additives, and their optimal wt% of dilution oil in MRG without the occurrence of sedimentation, was examined [32]. They discovered that the dilution oil altered the structure of the magnetizable particles in the grease medium, lowering the off-state viscosity MRG indirectly. Furthermore, the oil separation has formed when the oil dilution (castor and silicon oil) was exceeding than 10 wt%. Therefore, it was concluded that the addition of oil less than 10 wt% could produce a stable MRG. However, no studies have shown a correlation between viscosity and MR performance. Hence, this study examines the effect of MRG viscosity on the torque performance of an MR brake in various magnetic fields. In this investigation, two types of dilution oils with a fixed wt% of CIP were synthesized. The rheological properties such as apparent viscosity, shear stress and yield stress of MRG were investigated, and the torque performance of the MR brake was evaluated. To reduce the heat problem on the MR brake, which might affect transmission performance and transmission accuracy of MR devices, the study used a smaller range of current supply and a lower angular speed in the evaluation of torque performance of MR brake.

## 2. Materials and Methods

### 2.1. Fabrication of MRG

**Figure 1:**
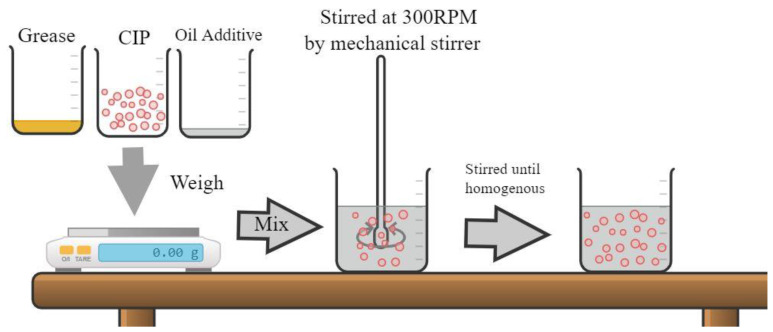
The schematic of the fabrication process of MRG samples.

In this study, soft magnetic particles (CIP), OM grade, in a spherical shape with diameter of 5 μm and density of 7.874 g/cm^3^, were purchased from BASF Germany. The CIP was selected for its high saturation magnetization and soft magnetic properties [33]. Meanwhile, commercial grease from NPC Highrex HD-3 grease Nippon Koyu Ltd., Osaka, Japan was chosen as a carrier medium. The grease density and the grease viscosity were 0.2 g/cm and 0.207 Pa.s, respectively. As additives in MRG, two types of dilution oil were used: kerosene with a density of 0.81 g/cm^3^ and hydraulic with a density of 0.88 g/cm^3^. Figure 1 illustrates the fabrication process of MRG samples with dilution oil. Initially, the grease was stirred for 5 min before mixed with 70 wt% of CIP by using a mechanical stirrer, (Multimix High Speed 1 Dispersed (HSD)). According to Mohamad et al. [22], MRG with 70% CIP had the maximum yield stress and relative MR effect of up to 52.7 kPa and 952.38%, respectively.

In another study conducted by Mohamad et al. [31], the addition of more than 10 wt% dilution oil would form unstable MRG and the potential of magnetic particles to settle was high, affecting the entanglement of fibrous structuration MRG. As a result, the MRG with a 10% oil dilution was predicted to perform optimally. The mixture of grease, CIP, and dilution oil was stirred for two hours at 300 rpm using a mechanical stirrer to produce the homogeneous MRG. A similar procedure was conducted with pure MRG, labeled as MRG 1. Meanwhile, other MRG samples were prepared with the dilution of hydraulic oil and kerosene oil, labeled as MRG 2 and MRG 3, respectively. The details of each sample are shown in Table 1.

### 2.2. Characterization

The magnetic properties and rheological properties of MRG samples were tested to determine the properties of MRG samples. The magnetic properties of MRG samples were evaluated using a Vibrating Sample Magnetometer (VSM) (Lakeshore, 7407 series). At room temperature, the samples were examined with a 1T maximum magnetic field. Meanwhile, the rheological properties were investigated using a parallel-plate rheometer (Anton Paar, Physica, MCR 302) under rotational mode. The application of an external magnetic field can change the rheological characteristics of MRG such as viscosity, shear stress, and yield stress. A volume of 1 mL of each MRG sample, measured by a syringe, was put into the base plate and the gap between the plate was set to 1 mm. The viscosity and shear stress were taken within the range of 0.1 to 100 s^−1^ and the magnetic field was set to zero by varying the current from 0 to 1.2 A with 0.4. Table 2 shows the DC magnetic field in terms of magnetic flux density for each increment of current. All the experiments were conducted at room temperature of 25 °C.

### 2.3. The Evaluation of the Diluted MRG in MR Brake

**Figure 2:**
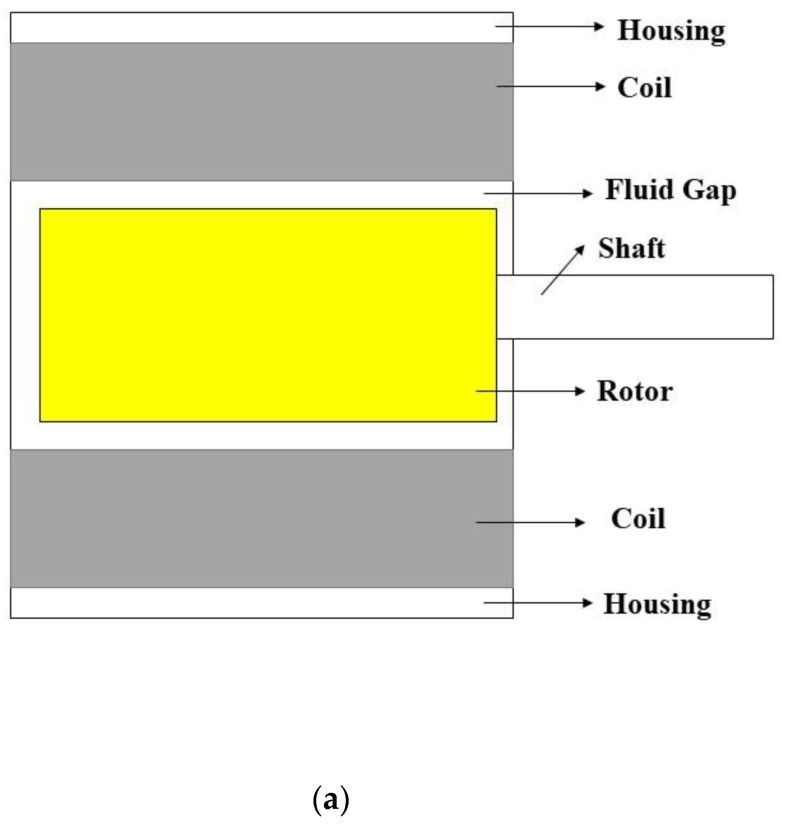

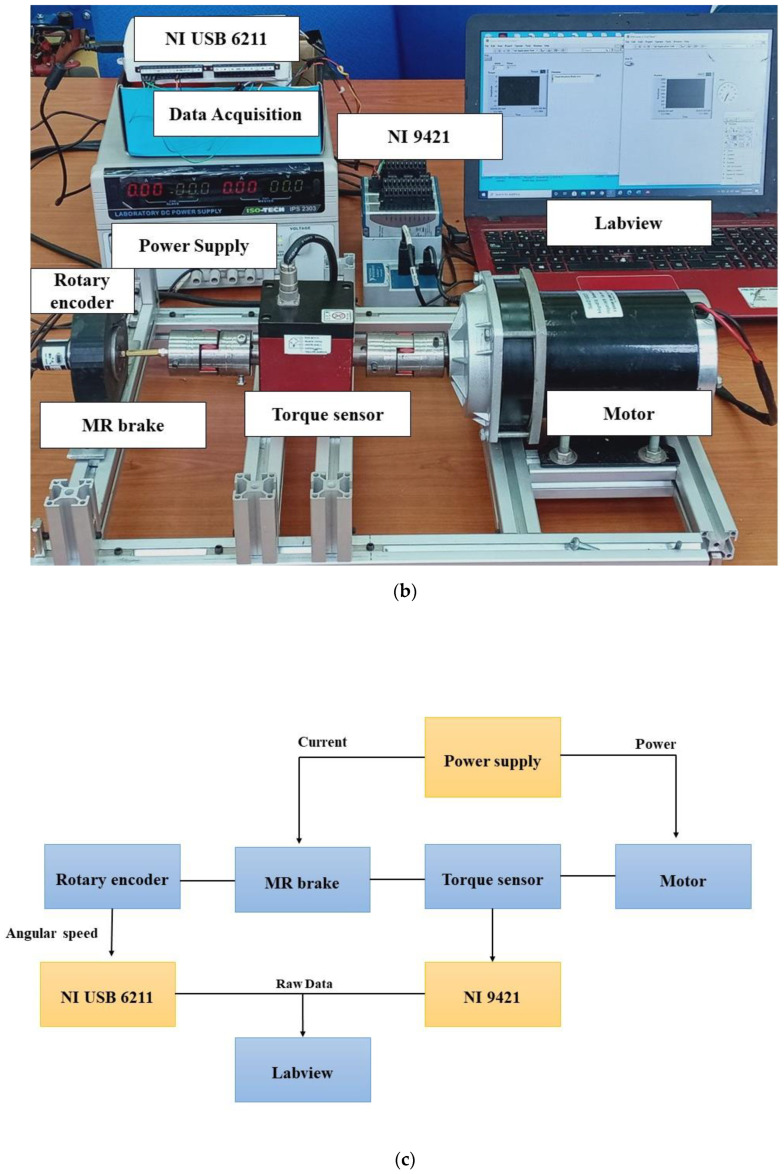
(**a**) Schematic drawing of MR brake, (**b**) experimental setup and (**c**) diagram chart of the test rig.

The performance of MRG samples was investigated to determine the relationship between the applied current and torque of the MR brake. Figure 2 demonstrates the experimental work setup. Figure 2a displays the configuration of MR brake, Figure 2b represents the experimental setup to evaluate the torque performances of MRG, while Figure 2c shows the diagram chart of the test rig.

A syringe was used to inject 0.8 mL of MRG sample into the MR brake’s fluid gap region. Then, the torque performance test was conducted by using shaft type rotary torque transducers or torque sensor (TCS-1000KC model) from CTAplus with capability from 5 to 1000 kgf.cm. This torque sensor, which consisted of a strain gauge bridge, could be used on low torque devices with input voltages of up to 10 V to determine the rotational torque produced by MR brake. As a moving source, a DC motor was used, which was powered by a power supply (ISO-TECH IPS-2303). The shaft from MR brake and shaft from motor were parallelly joined by using jaw coupling, which was suited for determining the continuous rotational torque. Then, the rotational speed was fixed to 40 rpm by employing an encoder (Cytron B 106). The rotating speed was limited to 40 rpm since greater speeds were produced unstable result owing to MR braking limitations. With a 0.4 A increment, currents ranging from 0 to 1.2 A were applied to the MR brake. The increment of current would generate higher braking torque by increasing the resistance between the rotor and carrier fluid in MR brake. When the current was increased over 1.2 A, however, the torque performance of the MRG became unstable owing to a heat issue in the MR brake. As a consequence, low current and low rotary were employed to reduce heat in the MR brake, which might influence transmission performance and accuracy of MR devices. The raw data generated by torque sensor were in analog signals, which may be harmful to the monitor equipment when directly analyzed. The data were then converted into signal conditioning by using National Instrument USB 6211 and National Instrument 9421 to ensure its safe and readable. In other words, the National Instrument 9421 was used to translate the raw data from torque sensor, while National Instrument USB 6211 for translating the raw data from encoder to ensure a constant rotary speed of MR brake. Both translated data were then displayed on the computer by using LabVIEW 2013 software.

## 3. Results

### 3.1. Magnetic Properties of MRG

**Figure 3:**
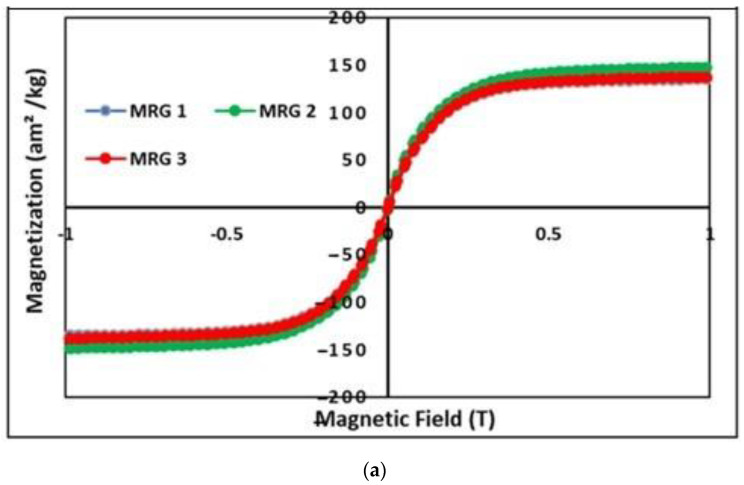

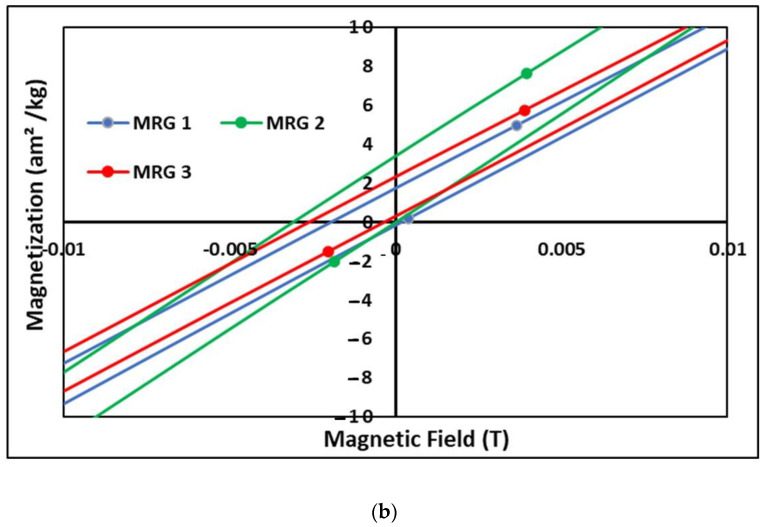
(**a**) The magnetization curve of MRG with different type of oil dilution, (**b**) enlargement of the hysteresis region at various magnetic fields.

Figure 3 depicts the magnetization saturation of MRG samples at room temperature, which were examined using VSM over a range of magnetic properties ranging from −1 T to 1 T.

Figure 3a depicts that the CIP performs extremely well in terms of soft magnetic properties at room temperature. When the magnetic field is raised, the magnetic flux density rises and eventually becomes saturated. The weight percentage of magnetic particles suspended in the grease strongly influences the magnetic property of MRG. As a consequence, even after dilution oil was added to MRG, the magnetization saturation was predicted to be almost the same. Despite this, the magnetization saturation of MRG 2 were found to be the greatest, at 148 am^2^/kg^−1^, followed by MRG 3 and MRG 1, with 138 am^2^/kg^−^^1^ and 135 am^2^/kg^−1^, respectively. According to the results, when hydraulic oil was applied, the magnetization saturation of MRG 2 increased by 9%. Meanwhile, MRG 3 has only increased by 2% in comparison to MRG 1. This occurred due to the MRG with dilution oil having a better CIP distribution in the suspending medium compared to pure MRG [22]. The addition of oil dilution aided in the mixing of CIP and grease medium, as well as the reduction of CIP distance in grease medium. With dilution oils present during on-state conditions, CIP in the grease medium with less mobility limitation has assisted in a better chain formation [31]. However, the difference in magnetization saturation between all samples was negligible. Consequently, it is reasonable to conclude that the addition of oil has no noticeable impact on the magnetization saturation of MRG. Figure 3b shows the tiny hysteresis loop curves obtained for each sample. However, when the enlargement at low magnetic field. The figure has showed an asymmetry behavior. This phenomenon probably occurred due to external field range which probably oriented by the direction of the electromagnet field. Moreover, the insufficient field received by the sample maybe one of the reasons for not symmetry.

### 3.2. Finite Element Method Magnetic (FEMM)

**Figure 4:**
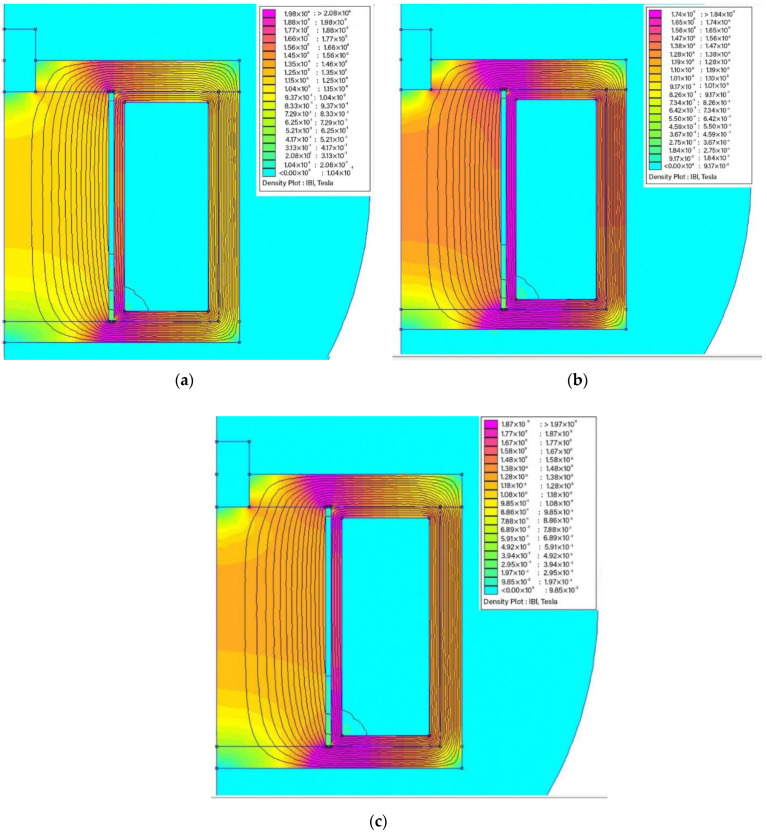
The magnetic fields distribution (**a**) 0.4 A, (**b**) 0.8 A, and (**c**) 1.2 A.

The magnetic flux pattern within the MR brake was investigated by using the open license software FEMM (Finite Element Methods Magnetics) Version 4.2. In the first phase, half of the MR brake’s longitudinal cross section was drawn in axisymmetric mode. Following the software library, it was followed by material assignment. If the program does not support non-standard materials, such as MR fluids, the magnetic permeability can be manually entered into the software. Following material assignment, the meshing process was initiated. The model could then be run, particularly by adding an electric current. During simulation, the current was specifically 0.4 to 1.2 A by intervals of 0.4 A. Figure 4 depicts the magnetic field distribution at driven currents of 0.4, 0.8 and 1.2 A. Based on the result, the magnetic field intensity increases as the electric current increases, as seen by the red color near the effective braking area.

**Figure 5:**
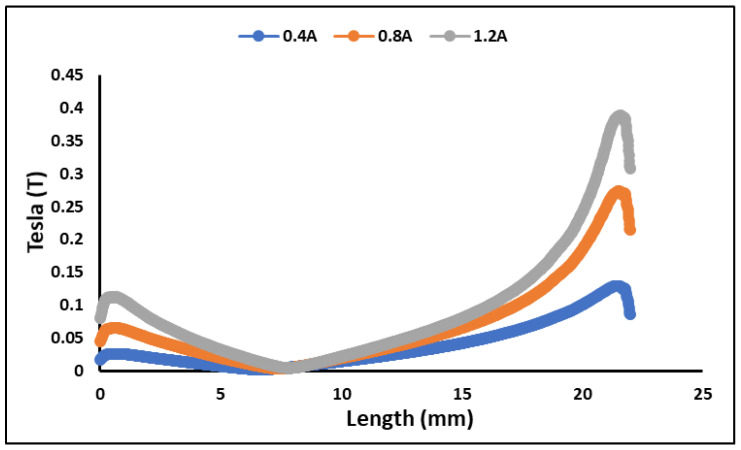
Flux Density value in MR brake’s fluid gap.

The flux density values at the fluid gap area are displayed in the Figure 5. The graph only displays the flux density for applied currents of 0.4, 0.8, and 1.2 A. Based on the result, the flux density varies from 0.02 to 0.11 T at top of the fluid gap and from 0.13 to 0.38 T at bottom of the fluid gap. It revealed that the magnetic flux is greatest around the coil area at the bottom of the casing. The difference in flux enhancement at low current, especially below at 0.4 to 0.8 A, is relatively large when compared to greater current (0.8–1.2 A). This is due to the fact that the obtained flux value closes the magnetization saturation of the magnetic materials. As a result, increasing the current will not result in a considerable improvement in the flux. Instead, applying more current raises the temperature of the copper wires, affecting the transmission performance and accuracy of MR devices. It can also be deduced that the radial area’s field may be modified to enhance the active surface area for further design improvement.

### 3.3. Rheological Properties of MRG

**Figure 6:**
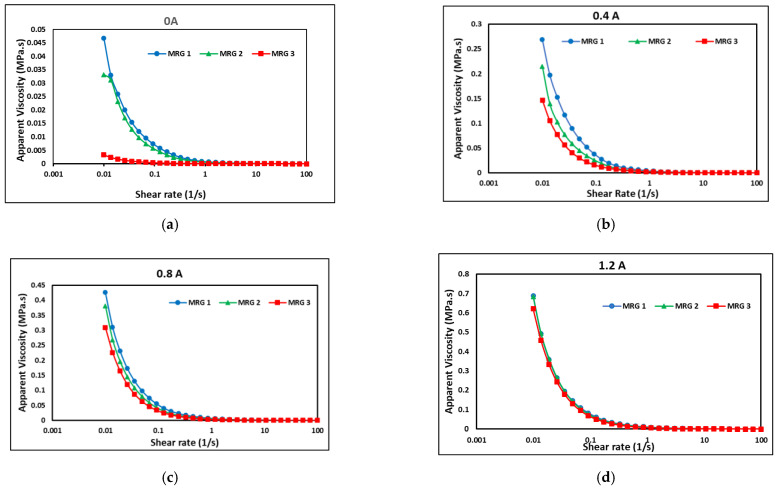
The viscosity of the MRG samples at (**a**) 0, (**b**) 0.4, (**c**) 0.8, and (**d**) 1.2 A of current.

Figure 6 depicts the apparent viscosity of MRG as a function of shear rate from 0.01 to 100 s^−1^ under various currents of 0, 0.4, 0.8, and 1.2 A.

The initial viscosity of MRG was observed to be lowered after dilution oil (hydraulic and kerosene) was added to the MRG. MRG 1 had the greatest initial viscosity of 0.0467 MPa.s, MRG 2 had 0.0348 MPa.s, and MRG 3 had the lowest initial viscosity of 0.00335 MPa.s. The result proved that the hydraulic and kerosene oils were effective in lowering the initial viscosity of MRG by 25% and 93%, respectively. However, due to the difference in oil viscosity, there was a significant difference in apparent viscosity between MRG 2 and MRG 3. Kerosene oil has a lower viscosity than hydraulic oil, resulting in a less viscous MRG. It was noted that the dilution oil in MRG reacted as a lubricant that loosen the fibrous entanglement structures of the grease with the influence of the shear rate. In fact, the MRG 3 result exhibited a lower apparent viscosity when compared to Tarmizi et al. [20], who succeeded to reduce the MRG starting viscosity by roughly 86% by adding cobalt ferrite. However, the viscosity of MRG 2 exhibited a significant shift in trend in Figure 6a. According to Schippa et al. [34], when the rheometer began to accelerate, particle collisions and fluid-induced lift-drag forces result in a considerable drop in the apparent viscosity of the mixture, and at the correct amount of stress, it may approach a steady state stress-strain condition.

In another finding, the apparent viscosity of all samples increased in the presence of a magnetic field, as shown in Figure 6. As the current raised to 0.4 A, the apparent viscosity of MRG 3 progressively rose to 0.147 MPa.s. Meanwhile, the apparent viscosity of MRG 1 and MRG 2 has increased to 0.269 MPa.s and 0.215 MPa.s, respectively, which was still greater than MRG 3. However, the difference in apparent viscosity between MRG 2 and MRG 3 was greatly reduced with the increment of magnetic field. The same trend happened when the current was increased to 0.8 A, the apparent viscosity of MRG 1 rose to 0.426 MPa.s, whereas the apparent viscosity of MRG 2 increased to 0.381 MPa.s, which was closely to MRG 1. Meanwhile, MRG 3 has an apparent viscosity of 0.310 MPa.s. The apparent viscosity difference between MRG 2 and MRG 3 was further reduced. Until 1.2 A of applied current, apparent viscosity of MRG 2 and MRG 3 has becoming nearly equal to MRG 1. According to Kim et al. [30], the addition of dilution oils to the MRG was not only capable to reduce the initial viscosity but also could improve the distribution of the CIP inside the grease with the result in the broadening of the inter-particle voids. Subsequently, the CIPs appeared to be less attached to the grease that led to sliding motion during the shearing process [31]. This occasion displayed that the addition of dilution oil in MRG has improved the reactivity between magnetic particle in MRG 3 with the increment of magnetic field and led the viscosity of MRG 3 to become almost same equal as other samples. As the applied magnetic field has increased, the dominance of inter-particle attraction over shear force has lowered particle distance. Despite the impact of shear rate, the restriction of CIP mobility within the grease caused to an increase in the apparent viscosity of MRG. In contrast, as the shear rate increased, the apparent viscosity of MRG samples decreased, indicated the pseudo-plastic behavior [35]. These trends are also known as a shear-thinning behavior of MRG [20]. This phenomenon was most likely caused by changes in the chain-like structure of CIPs as shear rate increased and grease characteristics changed. With the presence of magnetic field, CIPs produced a chain-like structure at a low shear rate. However, the MRG’s chain-like structure decreased owing to significant deformation with increasing shear rate, resulting in a decrease in apparent viscosity [33,35].

**Figure 7:**
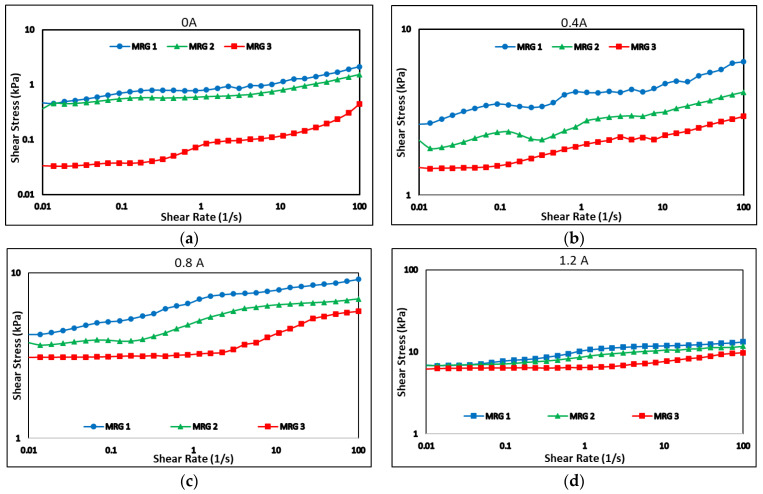
The shear stress of the samples at (**a**) 0, (**b**) 0.4 (**c**) 0.8 and (**d**) 1.2 A of current.

Figure 7 represents the dynamic shear stress of MRG samples as a function of the shear rate under the application of 0, 0.4, 0.8 and 1.2 A of current. At zero magnetic field, all samples exhibited non-Newtonian behavior, which was due to magnetic particle remnant magnetization and the intrinsic property of the grease [20,33,36]. However, when the shear rate increased, the shear stresses of the samples were unstable due to the random dispersion of particles and wall slip motion in the grease medium as well as plate shearing [20].

The presence of dilution oils in MRG 2 and MRG 3 have reduced the shear stress to 0.347 and 0.0335 kPa, respectively, in comparison with MRG 1 at 0.466 kPa. This decreasing trend happened for both off- and on-state conditions, indicated that the diluting oil acted as a lubricant to aid in the shearing action in MRG 2 and MRG 3. The shear stress trends became more stable as magnetic fields rose, owing to the formation of solid-like characteristics and chain structure perpendicular to the shear flow direction, which was aided by the dipole-dipole interaction between the CIPs. Furthermore, the shear stress has increased with the shear rate. According to Park et al. [33], as the shear rate increased, the entanglement of a fibrous structure in grease began to deteriorate, and the grease became less viscous. This condition has improved the motion of CIPs for a better chain formation. Furthermore, the samples exhibited Bingham behavior, which might be attributed to the formation of chain alignment by magnetic particle in the grease medium. As a result, it was possible to conclude that MRG exhibited Bingham behavior with non-vanishing yield stress in the presence and absence of a magnetic field. This yield behavior was attributed to the inherent properties of grease and the formation of the chain structure of magnetized CIP in MRG in presence of an external magnetic field [22,27].

**Figure 8:**
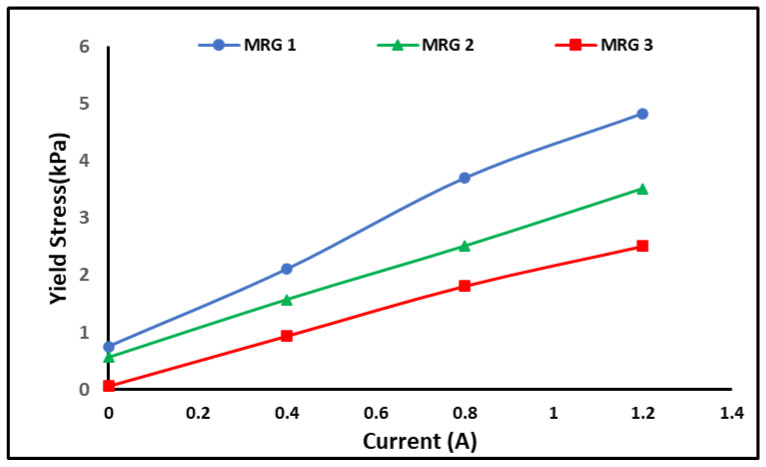
The yield stress of the MRG with different types of dilution oil under various applied currents.

In most MR applications including MR brake, the high yield stress would lead to a high braking torque [28]. The yield stress is an important parameter that contributes to the efficiency of MR devices. In general, extrapolating the shear stress to zero shear rates produces the yield stress level of MR grease. Without the stimulus of a magnetic field, the yield stress of MR grease is dominated by the grease properties as in the viscosity and shear stress tests [20]. Figure 8 shows the dynamic yield stress increases by the increment of the applied current of 0, 0.4, 0.8, and 1.2 A. As expected, the yield stress of the MRG samples was increased parallelly to the current applied. MRG 1 produced more off-state yield stress (0.751 kPa) than MRG 2 and MRG 3, which produced 0.562 and 0.0552 kPa, respectively. Meanwhile, at on-state condition, MRG 1 has the highest yield stress that was 9.151 kPa and followed by MRG 2 and MRG 3 with 8.279 and 6.609 kPa, respectively. By increasing the magnetic field, the dipole-dipole interaction was stronger and hindered the flow in which increased the yield stress values [33,37]. However, in the absence of any magnetic field stimulation, the yield stress of MRG was dominated by typical grease properties. The finding showed that the viscous properties and efficiency of the MRG were affected by the dilution oil.

### 3.4. Evaluation of Diluted MRG Performance in MR Brake

**Figure 9:**
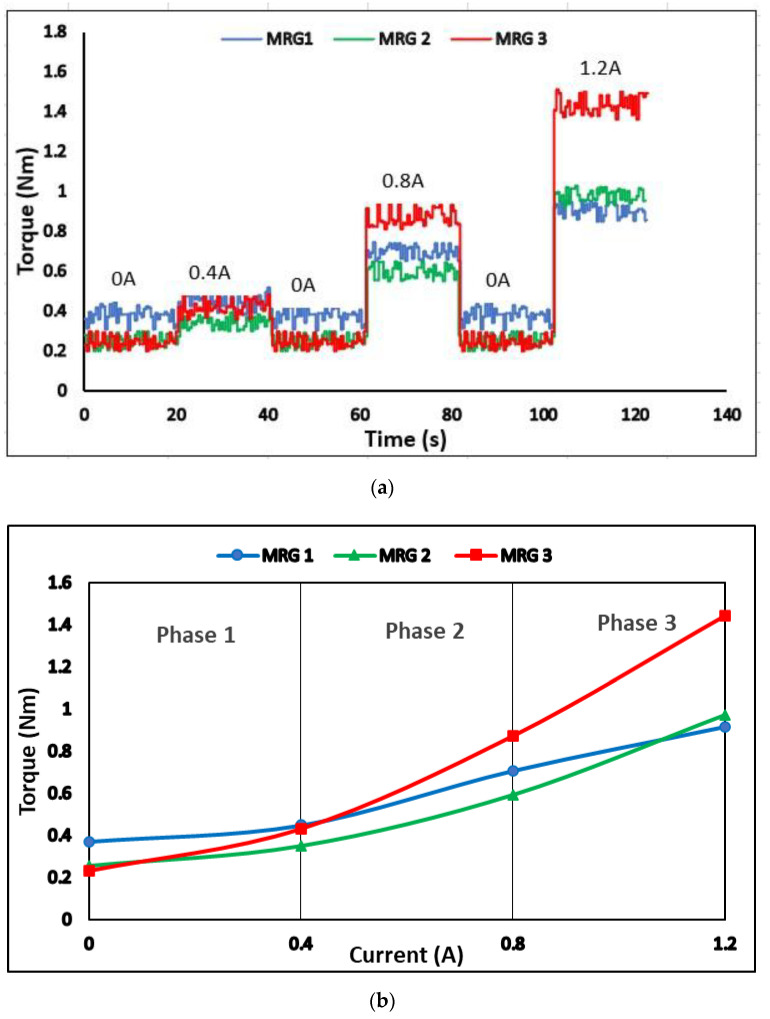
(**a**) Step response in relation to a 40 rpm and various current and (**b**) their average of torque under various of current.

In this study, the torque was adjusted in response to the various step input currents. Figure 9a depicts the torque step responses at rotary speed of 40 rpm in 140 s for all samples. At the on-state condition, the produced torque in the MR brake using MRG 3 was greater than the other samples. In brief, the braking torque was influenced by three main factors, magnetic field, viscosity of oil, and mechanical friction of the device [38]. For the first factor, the torque performance was affected by the field-dependent yield stress, where the shear stress restricted the rotation of the brake disk by controlling the magnetic field strength [39]. As seen in Figure 9, MRG 3 produced more torque at on-state condition. It was discovered that the restriction of shaft rotation was also increased due to the impact of utilizing high viscous properties. The addition of low viscosity kerosene, on other hand, has modified the base oil to thickener ratio, causing the viscosity of MRG 3 to drop dramatically. In other words, the low viscosity of dilution oil was assumed to severely damaged the fibrous structure of grease [40]. The physical breakdown of grease’s thickener structure happened when it was exposed to high mechanical stress or vigorous stirring [40,41,42]. Thus, the physical breakdown of the fibrous structure in MRG 3 was increased, as kerosene dilution oil has assisted the stirring process in the fabrication of MRG by improving the lubricating abilities of MRG. This phenomenon has significantly reduced the viscosity of MRG 3. Nonetheless, when the current was applied, this incident improved the CIP response in MRG. As a result, the significant viscoelastic pressures generated by the grease network in MRG 1 have hampered the orientation and alignment of the CIP chain structures [21]. Meanwhile, the kerosene dilution oil has improved the orientation and alignment of the CIP chain structure in MRG 3 as well as the time response since the reaction between CIP increases in on-state condition. In the meantime, a different torque trend was depicted for MRG 1 and MRG 2. MRG 2 exhibited a low torque performance for current below than 0.8 A which resulting by the hydraulic oil properties. However, when the current was increased to 1.2 A, the torque of MRG 2 was higher than MRG 1. The addition of hydraulic oil to grease is also thought to degrade the fibrous structure of the grease, but not as severely as kerosene oil. This phenomenon occurred because the hydraulic oil has a longer carbon chain, ranging from 15 to 50 carbon atoms, than kerosene oil, which has a carbon chain ranging from 10 to 16 carbon atoms. The higher the carbon number, the higher viscosity of oil [43]. Therefore, hydraulic oil has a higher viscosity than kerosene oil. The addition of hydraulic oil in MRG has slightly changed the ratio of base oil to thickener and reduced the viscosity of MRG 2.

The average torque performances under application of current were depicted Figure 9b. At phase 1, MRG 1 exhibited the highest torque value; 0.37 Nm at 0 A followed by MRG 2 and MRG 3 with 0.26 Nm and 0.23 Nm, respectively. This scenario was due to the high initial viscosity and mechanical friction which occurred in all samples prior to application of magnetic field. It was discovered that the addition of dilution oil that work as lubricant has reduce the friction force between the MRGs’ and MR brake [31,32,44]. In phase 2, the torque of MRG 3 was observed to overlapping with MRG 1 at current of 0.4A and surpass as the current reached to 0.8 A. Meanwhile, MRG 2 torque value was gradually increased, but remained lower than the torque value of MRG 1. It shows that MRG 3 with lowest viscosity has improved the mobility of the CIP in grease medium which improving the interaction between CIP as magnetic field was applied. As the magnetic field was increased, the braking torque also increased since the reaction between CIP in MRG 3 increased compared to MRG 1 and MRG 2. At phase 3 with the implementation of 1.2 A towards the MRGs’ samples, MRG 3 gained the highest torque value while MRG 2 has slowly increased and exceed MRG 1. In other words, the addition of hydraulic dilution oil has only slightly increased the braking torque of MRG 2 after 1.2 A. Although the viscosity MRG 2 has showed slightly lower compared to MRG 1, it can slightly increase the torque performances of MR brake. Overall, the torques performance of MRG 2 and MRG 3 increases to 0.97 Nm and 1.44 Nm, respectively, compared to 0.91 Nm gained by MRG 1.

**Figure 10:**
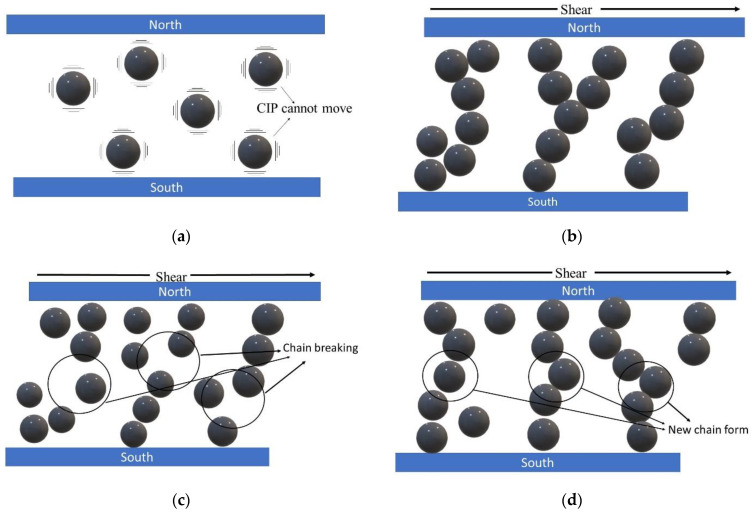
The illustration of CIP movement in shear mode-MR brake in (**a**) absence of magnetic field, (**b**) presence of magnetic field, (**c**) chain breaking, and (**d**) reformation of new chain.

Figure 10 shows an illustration of CIP movement in a shear mode MR brake. Figure 10a shows that in the absence of an external magnetic field, CIP was distributed randomly in the grease medium. When a magnetic field was presented, the particles quickly aggregate into chains that are oriented to the magnetic field and forming the chain structure as shown in Figure 10b. As shear force and current were applied to the samples continually, the process of breaking and re-forming new chains can be seen, as shown in Figure 10c,d. It was demonstrated that CIP mobility limitation caused by grease viscosity lowered chain formation strength, which directly reduced MRG 1 braking torque. Consequently, the behavior of chain formation in MRG 2 and MRG 3 has been improved with the presence of dilution oil viscous properties. As a result, CIPs in MRG 2 and MRG 3 have better inter-particle interactions and forming more structured and harder chain alignment. It can be concluded that addition of dilution oil in MRG samples was proven capable to improve the performance torque efficiency.

**Figure 11:**
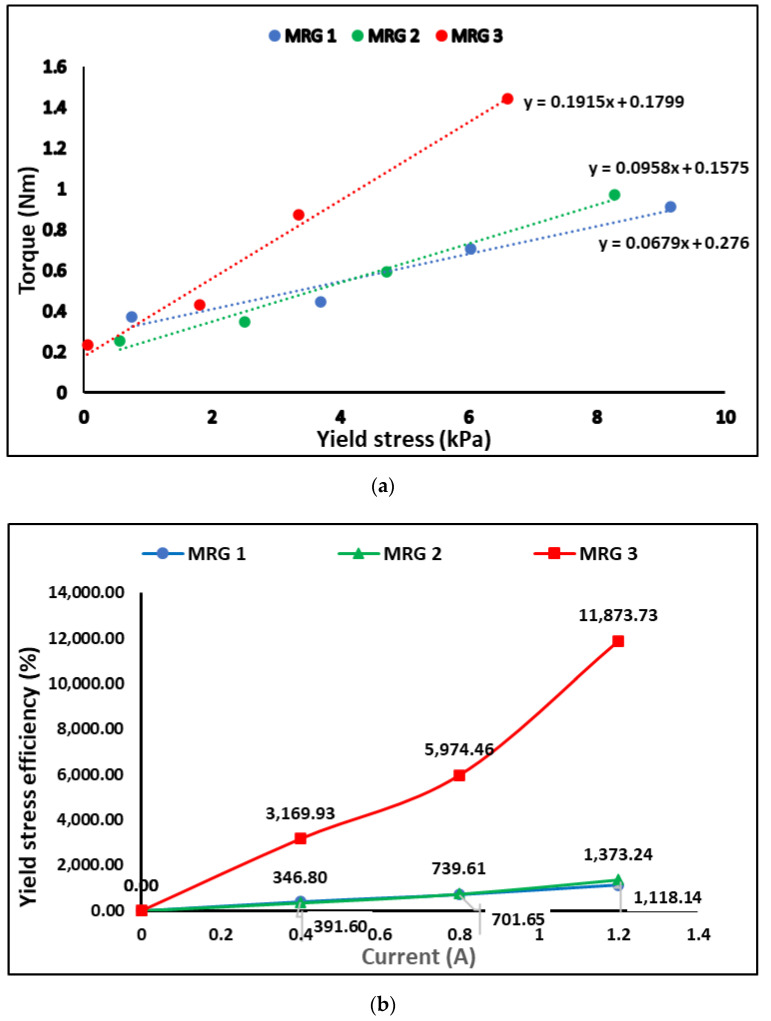

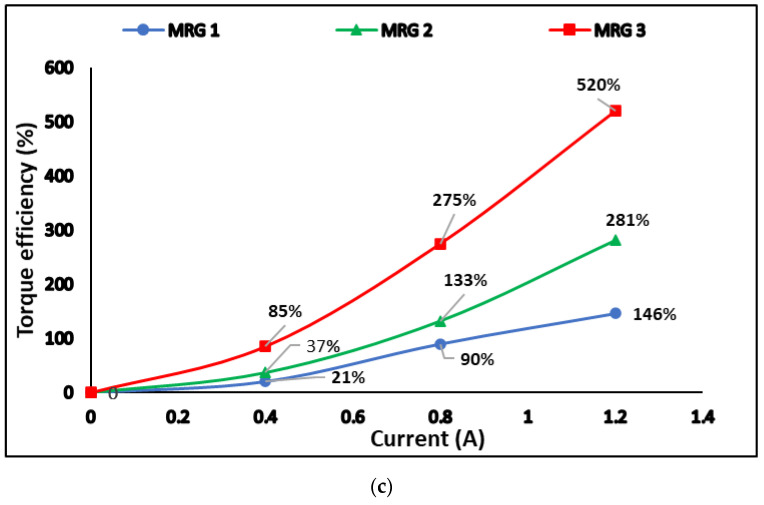
(**a**) The relationship between the yield stress of MRG and their braking torque and the percentage of the increment (**b**) yield stress of MRG and (**c**) braking torque of MRG.

Then, the yield stress obtained from rheometer and braking torque value from MR brake (refer Figure 11a) are investigated intensively for correlation in device performance perspective. Meanwhile, the efficiency of the yield stress and braking torque was evaluated to examine the increment of the yield stress and braking torque from off-state to on-state. The result then was depicted in Figure 11b,c. The percentage of the yield stress and torque efficiency has been calculated using the Equations (1) and (2), where $\tau_{y}$ is the yield stress of the MRG and T is the braking torque of MRG.

(1) $$The\;percentage\;of\;yield\;stress\;efficiency=\frac{\;\tau_{y\;\;on\;state}-\tau_{y\;\;Off\;state}\;}{\;\tau_{y\;\;off\;state}}\times 100\%$$

(2) $$The\;percentage\;of\;torque\;efficiency=\frac{T_{on-state}-T_{off-state}}{T_{off-state}}\times 100\%$$

Figure 11a showed that the yield stress of MRG has affected the torque performance of MRG in MR brake. In the off-state condition, MRG 3 has the lowest yield stress and generate the lowest braking torque performance followed by MRG 2 and MRG 1 in ascending order. It was predicted since MRG 1 had the maximum mechanical friction, including MRG with the MR brake’s inner surface, CIP-CIP, and CIP-grease. In general, mechanical friction between MRG and the MR brake’s inner housing and rotor has impacted the torque of the MR brake in off-state condition. The torque was increased due to the resistance of carrier fluid that flows in grease medium, which restricted the rotation of the shaft [45,46]. At the same time, the viscous friction between the MRG and MR brake also occurred. The rotation of the MR brake has increased the shear stress in carrier fluid and forced the CIP to move in the grease, subsequently, increased the collision between CIPs and CIP with grease medium. Expectedly, the addition of oil resulting on improving the lubrication of the MRG by reducing the mechanical friction. Aside from that, MRG with oil addition produced greater torque performance than MRG 1 with yield stress increase.

The efficiency of the yield stress and braking torque of MRG can be observed in Figure 11b,c. MRG 3’s yield stress efficiency has grown 3170%, while torque efficiency has increased 85% at 0.4 A. Both yield stress and torque efficiencies were much greater compared to MRG 1, which only generated 21% of the torque increment with 392% of yield stress. However, MRG 2 with dilution of hydraulic oil can generated 37% of torque efficiency which is higher compared to MRG 1 when the yield stress efficiency of MRG 2 increased to 347%. At 0.8 A, the torque produced by MRG 3 was 275% and the yield stress performance increased to 5974%. As the current increased, the performance disparity between all samples widened. For MRG 2, the yield stress increased up to 740% and the braking torque has increased almost 133%. Meanwhile, MRG 1 with high viscous properties has the lowest yield stress efficiency; 90% and torque efficiency; 702%. A similar phenomenon also observed at current applied of 1.2 A. The trend of yield stress and braking torque efficiency of MRG 3 was higher than MRG 2 and MRG 1. The great improvement of yield stress and torque performance of MRG 3 owing to viscous and friction properties on the application of magnetic field. As the magnetic field was applied, the CIP combine to form chain structures aligned with the magnetic flux direction. The bond strength of the chain formation may be constantly adjusted by the application of magnetic field [38]. The orientation of the CIP in the chain structures causes an increase in yield stress, which indirectly improves the torque performances of the MRG. At high magnetic fields, the alignment of the CIPs in MRG 3 has a better arrangement compared to other samples, resulting in stronger structures, fewer voids and better inter-particle interactions. Therefore, the yield stress efficiency and braking torque efficiency was expected to improve as the viscosity of grease was reduced. Compared to MRG 3, the yield stress efficiency and torque efficiency of MRG 2 and MRG 1, which have higher viscosity, are influenced by mechanical friction that occurs in grease medium. It has been proved that the fibrous structure of the grease was affected by the dilution oil. Obviously, in MRG 3, with addition of kerosene oil, the braking torque has enhanced the viscous properties and mechanical friction of MRG in the on-state condition. According to the findings, the braking torque of MR brake utilizing MRG can be controlled through the yield stress.

## 4. Conclusions

The torque performances of MRG with dilution of kerosene and hydraulic oils in MR brake has been evaluated. The results showed that adding oil dilution can lower the viscosity of MRG. However, the addition of oil has also reduced shear stress and yield stress of the MRG not only in off-state condition but also in on-state condition. However, they outperformed pure MRG in terms of torque performance (MRG 1) when magnetic field was applied. With the absence of magnetic field, the finding shows the initial torque of MRG 1 was the higher than MRG with dilution oil. The high viscosity in the grease medium has increased the viscous properties and mechanical friction in MR brake and generate a higher torque. Nonetheless, in the presence of a magnetic field, the torque of MRG 3 was higher than the other two samples which 1.44 Nm, followed by MRG 2 at 0.97 Nm, and MRG 1 at 0.91 Nm. This occurrence demonstrated that dilution oil improved the rheology properties of MRG, resulting in greater dispersion of CIP in MRG and less restriction of CIP movement in MRG. Thus, when the current was applied, the reaction between CIP to form a chain-like structure was improved in both MRG with dilution oil compared to pure MRG. As a result, this study was significant for improving the torque performance of MRG brakes by employing MRG with dilution oil, particularly kerosene oil, and revealed the correlation between MRG rheological properties and torque performance in MR brakes.

## Figures and Tables

**Table 1:** The composition of the sample of MRG with a different types of oil dilution.

Sample	Grease (wt%)	CIP (wt%)	Hydraulic Oil (wt%)	Kerosene Oil (wt%)
MRG 1	30	70	-	-
MRG 2	20	70	10	-
MRG 3	20	70	-	10

**Table 2:** Magnetic flux density of MRG.

Samples	Magnetic Flux Density (T)			
	Current (A)			
	0	0.4	0.8	1.2
MRG 1	0	0.082	0.168	0.253
MRG 2	0	0.081	0.167	0.251
MRG 3	0	0.074	0.153	0.231

## Data Availability

The raw/processed data required to reproduce these findings cannot be shared at this time as the data also form part of an ongoing study. In future, however, the raw data required to reproduce these findings will be available from the corresponding authors.
